# Functional Nutrition Literacy and Healthy and Sustainable Eating Efforts Across Diverse Populations

**DOI:** 10.1111/jhn.70355

**Published:** 2026-10-18

**Authors:** Alejandra Jáuregui, Edna Jacqueline Ramírez‐Contreras, Kathia Larissa Quevedo Torres, Alejandra Contreras‐Manzano, Christine M. White, Laura Patricia Arellano‐Gómez, Simón Barquera, David Hammond, Rachel E. Davis, James F. Thrasher

**Affiliations:** ^1^ Area of Nutrition Surveillance and Physical Activity, Center for Health and Nutrition Research Instituto Nacional de Salud Pública Cuernavaca Morelos Mexico; ^2^ Epidemiological Methods and Etiological Research Leibniz Institute for Prevention Research and Epidemiology ‐ BIPS Bremen Free Hanseatic City of Bremen Germany; ^3^ School of Public Health Sciences University of Waterloo Waterloo Ontario Canada; ^4^ Department of Psychology, Education and Health, ITESO Jesuit University of Guadalajara Guadalajara Jalisco Mexico; ^5^ Department of Health Promotion, Education and Behavior, Arnold School of Public Health University of South Carolina Columbia South Carolina USA

**Keywords:** dietary behavior, health literacy, Mexican Americans, Mexico, nutrition literacy, sustainable diets

## Abstract

**Introduction:**

Understanding how nutrition literacy influences individuals' efforts to adopt healthier and more sustainable dietary patterns is essential for informing effective public health strategies. Mexico and the United States have recently implemented several nutrition policies to promote healthier food choices within distinct social and food environments. We aimed to examine the association between functional nutrition literacy and healthy and sustainable eating efforts among Mexican Americans, non‐Latino Whites in the US, and Mexicans.

**Methods:**

Online surveys were conducted among adults in Mexico (*n* = 5779) and the US (Mexican Americans = 3343; non‐Latino Whites = 3674) as part of the International Food Policy Study 2021. Nutrition literacy was assessed using six questions based on country‐specific nutrition facts labels. Efforts to modify diet across food categories were used to construct indicators of healthy and sustainable eating aligned with international guidance. Logistic regression models estimated associations between functional nutrition literacy and dietary efforts, stratified by population group.

**Results:**

Healthy and sustainable eating efforts were similar between Mexican Americans and Mexicans (≈50%–80%), but lower for non‐Latino Whites (≈37%–65%), despite lower literacy levels in the former groups. Higher nutrition literacy was associated with greater likelihood of engaging in most dietary efforts across all populations, including increasing fruit, vegetable, and whole grain intake, and reducing consumption of processed foods, sugary drinks, and nutrients of concern (AORs = 1.07–2.73). Some associations were stronger among Mexican Americans and Mexicans (*p* < 0.008).

**Conclusions:**

Functional nutrition literacy is positively associated with healthy and sustainable eating efforts, supporting its role as a target for public health strategies alongside population‐level interventions.

## Highlights

- This study compared the association between functional nutrition literacy and dietary efforts across Mexican and US populations.
- Healthy and sustainable eating efforts were similar among Mexican Americans and Mexicans (≈50%–80%), but lower among non‐Latino Whites (≈37%–65%).
- Higher nutrition literacy was associated with most dietary efforts across the three populations.
- The strength of some associations varied across population groups, with stronger associations among Mexican Americans and Mexicans.
- Functional nutrition literacy may support public health strategies across diverse populations.

## 1 Introduction

Dietary guidelines have traditionally aimed to promote adequate nutrition, health, and well‐being, primarily by preventing malnutrition and chronic disease [1]. More recently, and aligned with the United Nations' Sustainable Development Goals [2], attention has expanded to include the environmental impact of food systems, emphasizing the need to shift current dietary patterns toward more sustainable diets that protect the planet while ensuring access to healthy and nutritious foods for all [3]. These healthy and sustainable diets prioritize unprocessed or minimally processed foods, locally produced foods with lower environmental impact, appropriate caloric intake, and limited intake of saturated fats, added sugars, and sodium [4].

Following the implementation of the North American Free Trade Agreement in 1994, food systems in the neighboring countries of Mexico and the United States (US) have been increasingly integrated and characterized by similar dietary and chronic disease patterns [5, 6]. In both countries, obesity is a leading public health issue, contributing substantially to the burden of type 2 diabetes, cardiovascular disease, and other diet‐related conditions [7, 8]. Dietary patterns in Mexico and the US are characterized by high consumption of refined grains, dairy, added sugars, and animal‐based proteins and low intake of vegetables, fruits, and legumes [9, 10]. These dietary patterns have important environmental implications, including increased greenhouse gas emissions and greater land and water use associated with diets high in animal‐source foods and processed products [11, 12]. Furthermore, Mexican‐Americans, who represent about two‐thirds of US Latinos or around 40 million—are the ethnic group with the largest dietary carbon footprint in the US [12]. In response, both countries have implemented various policies and programs. Mexico implemented a tax on sugary beverages in 2014 and, in 2020, instituted front‐of‐package warning labels [13], and broad public health campaigns promoting the consumption of “natural” (e.g., whole/minimally processed foods) and traditional Mexican foods. In 2020, the US nutrition facts label was updated to highlight caloric content in large bold font, and to display added sugars [14]. The 2020–2025 Dietary Guidelines for Americans recommended eating fruits, vegetables, whole grains, and protein, and limiting foods and beverages higher in added sugar, sodium and saturated fat [15].

Implementing healthy and sustainable diets requires more than dietary guidelines and recommendations, access to food or exposure to nutrition policies. It also requires individuals to have the knowledge, skills, and capacity to interpret and act on nutrition guidance. These characteristics are captured by the broader concept of nutrition literacy, a nutrition‐specific form of health literacy that encompasses the skills needed to engage with and use nutrition information in everyday life [16]. Nutrition literacy has been conceptualized as a multidimensional continuum, encompassing functional, interactive, and critical dimensions [17, 18, 19]. Functional nutrition literacy refers to the basic skills needed to obtain, understand, and use nutrition information in everyday decision‐making, including interpreting information presented on food labels. Interactive nutrition literacy involves more advanced cognitive and interpersonal skills to communicate about nutrition and use social and community resources to support healthy choices, whereas critical nutrition literacy involves the ability to critically evaluate nutrition information and recognize the broader social, commercial, and environmental factors that shape dietary behaviors. Within this framework, functional nutrition literacy may represent an important foundation for dietary decision‐making by enabling individuals to understand nutrition recommendations, interpret nutritional information, compare food options, and translate these processes into dietary choices. Thus, nutrition literacy may facilitate informed food‐related decision‐making and support healthier dietary practices [20, 21], although translating nutrition‐related knowledge and skills into dietary behaviors may also depend on both individual capacities and structural and contextual factors [21]. Limited nutrition literacy has been associated with reduced ability to make informed food choices and less healthy dietary habits [22, 23, 24, 25, 26]. However, evidence regarding the association between nutrition literacy and dietary behaviors remains inconsistent [20, 21], with limited evidence specifically addressing healthy and sustainable food choices. This highlights the need for further research to understand the role of functional nutrition literacy in supporting healthy and sustainable dietary practices.

In particular, most research examining nutrition literacy in relation to sustainability has been conducted in countries such as Australia, Canada, and the US and has focused primarily on conceptual frameworks or educational approaches [27], with relatively few empirical studies assessing behavioral associations. Studies from Turkey reported that higher diet‐related health literacy was positively associated with environmentally responsible food choices, such as the consumption of local and seasonal foods, avoiding food waste, reducing meat consumption, following a low‐fat, healthy diet that provides balanced nutrition [28, 29] and lower consumption of ultraprocessed products [30]. However, no studies to date have examined these relationships in Mexico or compared associations across ethnic groups in the US [27]. Cross‐national evidence is also scarce: it is unknown whether similar literacy levels translate into different behaviors across distinct sociocultural or policy environments.

Given the cultural, economic, and demographic ties between Mexico and Mexican‐heritage populations in the US, understanding how nutrition literacy relates to healthy and sustainable eating efforts across these groups can help clarify the extent to which individual‐level skills support dietary behavior change within different sociocultural and policy contexts, and informing effective public health strategies. The ability to interpret and apply nutrition label information may support healthier, potentially more sustainable dietary efforts by enabling individuals to compare products, monitor intake of nutrients such as added sugars and saturated fat, and make informed decisions within increasingly complex food environments. Social and policy environments differ markedly between Mexico and the US, as do exposure to labeling policies, educational systems, food access, and socioeconomic conditions [31, 32]. These contrasts offer a valuable opportunity to examine whether similar levels of literacy operate differently across contexts.

Using cross‐sectional data from the International Food Policy Study (IFPS), this study aimed to compare the association between functional nutrition literacy and self‐reported healthy and sustainable eating efforts among US Mexican American adults, US non‐Latino White adults, and adults in Mexico.

## 2 Methods

### 2.1 Study Design

Data were drawn from the Mexico and US arms of the 2021 IFPS, a repeated cross‐sectional online survey of adults aged 18–100 conducted annually in November–December. Detailed methods have been published elsewhere [33].

### 2.2 Participants and Recruitment

Participants were recruited in Mexico and the US through the Nielsen Consumer Insights Global Panel and its partner panels, using probability and non‐probability sampling methods. Email and dashboard invitations with unique links were shared with a random sample of panelists in both countries based on known population proportions for males and females across four age groups (18–29, 30–44, 45–64, and ≥ 65 years). Those known to be ineligible were not invited. Additional oversamples of individuals with lower educational attainment (i.e., high school education or less) in Mexico and with Mexican heritage in the US were recruited through Qualtrics and their partner panels. Surveys were self‐administered online in Spanish in Mexico and in English or Spanish in the US. All participants provided informed consent before beginning the survey. Participants were compensated according to each panel's incentive system (e.g., points, monetary rewards, or prize draws).

For the present analyses, participants were categorized into three population groups according to their Mexican heritage: (1) Mexican Americans (participants who self‐reported Mexican heritage and living in the US), (2) Non‐Latino Whites, and (3) Mexicans (all participants living in Mexico). After excluding respondents with missing data on any questions of interest (*n* = 719, 5.6%), the final analytic sample included 12,079 participants (Mexican Americans: 3342; Non‐Latino Whites: 2957; Mexicans: 5779).

The study received ethics clearance through a University of Waterloo Research Ethics Board (REB#30829), the University of South Carolina Institutional Review Board, and the National Institute of Public Health of Mexico (CI: 1641).

### 2.3 Measures

Functional Nutrition Literacy. Functional nutrition literacy was assessed with six items adapted from the Newest Vital Sign (NVS) [23, 34]. The original NVS was developed in English and Spanish and validated for use in the US as an interviewer‐administered health literacy assessment tool [23, 35]. The items used in the IFPS were further adapted to enable use in a self‐administered online survey without auditory narration and utilized open‐ended responses. The questions were further tailored for use in Mexico, including the development of a Mexico‐specific nutrition facts label image. Unlike the original NVS version, the adapted measures used in the IFPS required respondents to read the questions and write answers in open‐ended text fields. Participants were shown an image of a country‐specific nutrition facts label for a container of ice cream and answered six questions that evaluated numeracy (math‐based calculations), prose literacy (reading and applying label information), and document literacy (understanding label content). Based on the number of correct responses, individuals were classified into three literacy levels: scores of 0–1 indicated a high likelihood of limited literacy; scores of 2–3 indicated the possibility of limited literacy; and scores of 4–6 indicated a high likelihood of adequate literacy.

Self‐reported Healthy and Sustainable Eating Efforts. Participants were asked “*Have you made an effort to consume more or less of the following in the past year?,”* which was followed by a list of 22 diet modification efforts. Response options included “consume less,” “consume more,” “no effort made,” “don't know,” or “refuse to answer.” For each item, responses consistent with healthy and sustainable dietary guidelines or regional front‐of‐pack labeling systems (i.e., consuming more of recommended foods or consuming less of foods recommended for limitation) [3, 36, 37] were coded as 1, while all other responses (“No effort made” or the inverse response) were coded as 0. “Don't know” and “refuse to answer” responses were treated as missing.

To summarize healthy and sustainable eating efforts, the 22 items were grouped into categories representing efforts to modify diet in key domains [1]: efforts to increase fruit and vegetable intake (1 item) [2]; efforts to increase plant‐based protein sources (1 item) [3]; efforts to increase whole grain consumption (1 item) [4]; efforts to reduce intake of all meats, red meat, and dairy (3 items) [5]; efforts to reduce ultra‐processed food intake (1 item) [6]; efforts to reduce intake of sugary beverages and 100% fruit juice (2 items) [7]; efforts to increase protein and fiber consumption (2 items) [8]; efforts to reduce calories, total fat, saturated fat, added sugar, and sodium intake (5 items) [9]; efforts to limit artificial and natural low‐calorie sweeteners (2 items); and [10] other efforts related to consumption of locally grown, organic, ethically sourced, or low greenhouse gas emissions foods (4 items). For each category representing more than one item, summed values were recoded as 0 (no effort made/negative effort) or 1 (any/positive effort).

Covariates. Covariates assessed included age in years, sex at birth (male, female), and educational attainment (categorized as low, medium, or high in accordance with country‐specific criteria). Perceived income adequacy was also assessed with the question, “*Thinking about your total monthly income, how difficult or easy is it for you to make ends meet?”* [38] Responses were recategorized as “Very difficult or difficult,” “Neither difficult nor easy,” “Easy or very easy.” Nutrition knowledge was assessed with the single question, “*How would you rate your nutrition knowledge?*” (recoded as “Not at all or a little knowledgeable,” “Somewhat knowledgeable,” “Very or extremely knowledgeable”). Food security was assessed using the USDA Adult Food Security Survey Module [39], which includes 10 items covering adult food access hardships (items HH1 to AD5a). Respondents were classified into standard food security categories (food secure, low food security, very low food security) based on the number of affirmative responses.

### 2.4 Data Analysis

All analyses were weighted using post‐stratification sample weights constructed using a raking algorithm with national population estimates for sex, age, region, ethnicity, and (US only) education, then rescaled to each country's analytic sample size. Estimates reported are weighted unless otherwise specified.

Weighted descriptive statistics summarized the sample characteristics and functional nutrition literacy levels for each population (Mexican Americans, non‐Latino Whites, and Mexicans). Differences between populations were assessed using Pearson Wald tests adjusted for the survey design, and all results are reported as percentages.

Percentages for each self‐reported healthy and sustainable eating effort across the three populations were estimated using logistic regression models that pooled data from all three populations and adjusted for covariates. Adjusted percentages for each population were obtained from model‐based predictions, and the results were graphed. To assess differences across populations, multiple comparisons were conducted by specifying different reference categories (i.e., Mexico vs. Mexican Americans vs non‐Latino Whites).

Associations between functional nutrition literacy and self‐reported dietary modification effort categories were tested using separate logistic regression models for each dietary effort category, adjusting for covariates. Initial pooled models including all three populations incorporated interaction terms between nutrition literacy and population group (Mexican Americans, non‐Latino Whites, and Mexicans). When no statistically significant interaction was detected (*p* ≥ 0.01), pooled estimates were reported. When significant interactions were identified (*p* < 0.01), population‐specific adjusted models were used to examine heterogeneity in associations. Pooled estimates are shown for all outcomes for completeness; interpretation focused on population‐specific estimates only when statistically significant interactions were observed.

Analyses were conducted in Stata 19. Significance was set at *p* < 0.01 to account for the large sample sizes and multiple models and comparisons across groups.

## 3 Results

The sociodemographic characteristics of participants are presented in Table 1. Pearson Wald tests indicated differences among the three populations for all sociodemographic variables except sex.

**Table 1:** Sample characteristics. International Food Policy Study, 2021 (*n* = 12,079).^a^

	Mexican Americans (*n* = 3343)	Non‐Latino Whites (*n* = 2957)	Mexicans (*n* = 5779)	
	% (99% CI)	% (99% CI)	% (99% CI)	*p*‐value^b^
Age category				< 0.001
18–29 years	29.5 (27.3, 31.7)	12.5 (10.9,14.3)	27.9 (25.9, 30.0)	
30–44 years	37.1 (34.7, 39.7)	21.7 (19.6, 23.9)	31.0 (28.8, 33.3)	
45–59 years	26.6 (24.4, 29.0)	26.0 (23.7, 28.4)	28.2 (26.1, 30.5)	
60+ years	6.8 (5.5, 8.3)	39.9 (37.4, 42.5)	12.8 (11.0, 15.05)	
Sex				0.776
Male	47.2 (44.6, 49.7)	48.1 (45.5, 50.7)	48.0 (45.5, 50.4)	
Female	52.8 (50.3, 55.4)	51.9 (49.3, 54.5)	52.0 (49.6, 54.5)	
Education level				< 0.001
Low	68.5 (66.3, 70.5)	51.4 (48.8, 54.0)	74.8 (72.9, 76.6)	
Medium	10.7 (9.7, 11.8)	9.9 (8.9, 11.1)	10.4 (8.9, 12.2)	
High	20.8 (19.0, 22.8)	38.6 (36.2, 41.1)	14.8 (13.8, 15.9)	
Income adequacy				< 0.001
Very difficult or difficult	34.0 (31.5, 36.5)	22.4 (20.2, 24.8)	47.2 (44.8, 49.7)	
Neither easy nor difficult	39.2 (36.8, 41.7)	28.2 (25.9, 30.7)	41.3 (38.9, 43.7)	
Easy or very easy	26.9 (24.7, 29.1)	49.4 (46.8, 52.0)	11.5 (10.2, 13.0)	
Nutrition Knowledge				< 0.001
Not knowledgeable	33.3 (30.9, 35.8)	39.6 (37.1, 42.3)	45.2 (42.7, 47.6)	
Somewhat knowledgeable	43.0 (40.5, 45.6)	39.9 (37.4, 42.5)	45.5 (43.1, 48.0)	
Knowledgleable	23.7 (21.6, 25.9)	20.4 (18.5, 22.6)	9.3 (8.1, 10.7)	
Food security				< 0.001
Very low food security	28.18 (25.9, 30.57)	15.6 (13.8, 18.0)	27.02 (24.86, 29.29)	
Low food security	20.4 (18.43, 22.53)	10.8 (9.3, 12.6)	24.67 (22.62, 26.84)	
Food secure	51.42 (48.87, 53.96)	73.6 (71.1, 75.9)	48.32 (45.88, 50.76)	
Language use				< 0.001
English	84.88 (82.87, 86.68)	98.6 (97.8, 99.2)	0 (0, 0)	
Spanish	15.12 (13.32, 17.13)	1.4 (0.8, 2.2)	100 (0, 100)	
Functional nutrition literacy				< 0.001
High likelihood of limited literacy	37.6 (35.13, 40.14)	21.4 (19.4, 23.7)	45.54 (43.11, 47.99)	
Possibility of limited literacy	25.26 (23.13, 27.51)	21.9 (19.8, 24.2)	32.03 (29.79, 34.34)	
High likelihood of adequate literacy	37.14 (34.74, 39.61)	56.7 (54.0, 59.3)	22.44 (20.48, 24.52)	

^a^ Weighted estimates.

^b^ P‐values estimated with Pearson Wald tests across all three populations.

Overall, Mexican Americans (range reporting efforts: 51.3%–77.4%) and Mexicans (range: 43.9%–78.5%) reported similar percentages of healthy and sustainable eating efforts as each other and at higher percentages than non‐Latino Whites (range: 39.8%–67.4%, *p* < 0.01) (Figure 1). The two exceptions to this pattern were for efforts to consume more protein and fiber, and efforts to eat more organic, ethically sourced, or environmentally friendly foods. For these categories, Mexican Americans reported the highest engagement among the three groups (*p* < 0.01).

**Figure 1:**
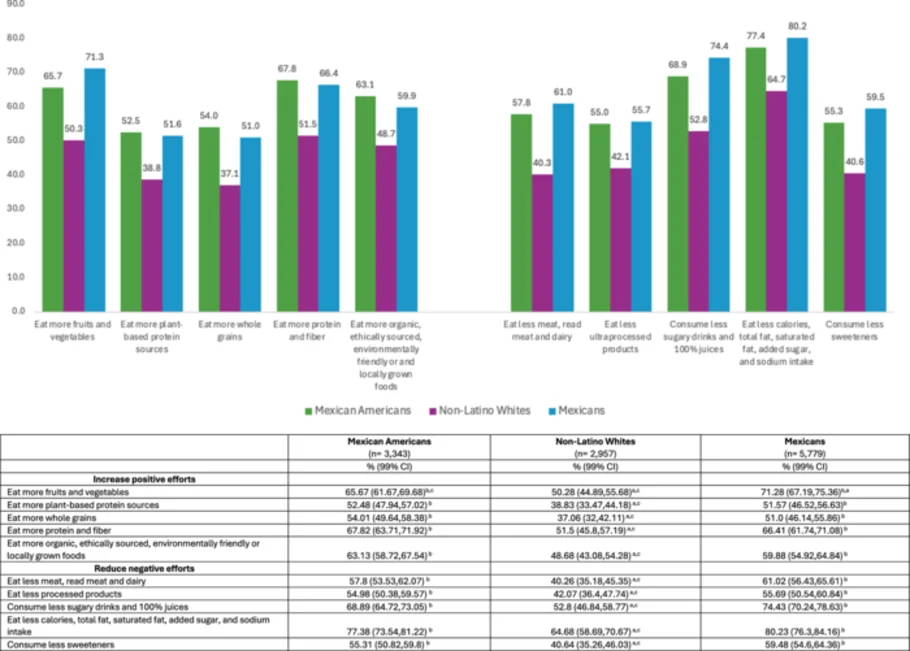
Adjusted* percentage of healthy and sustainable eating efforts among Mexican Americans, non‐Latino Whites, and Mexicans. International Food Policy Study, 2021 (*n* = 12,079). *Percentages adjusted by age, sex, education level, income adequacy, food security, and language use; a significantly different to Mexican Americans; b significantly different to non‐Latino Whites; c significantly different to Mexicans.

Models including all three populations indicated that having higher levels of functional nutrition literacy was consistently associated with greater odds of reporting to engage in multiple dietary efforts (Table 2). These included efforts to eat more fruits and vegetables (vs. a high likelihood or possibility of limited literacy; range of AORs: 1.36–1.43) and whole grains (vs. limited literacy: AOR = 1.22, 99% CI: 1.03, 1.45), as well as efforts to eat less meat, red meat or dairy (vs. possibility of limited literacy: AOR = 1.30, 99% CI: 1.09, 1.55), and calories, fat, saturated fat, added sugar and sodium (vs. high likelihood of limited literacy; range of AORs: 1.46–1.86). No interactions by population group were observed for these dietary efforts (all interaction p‐values > 0.01).

**Table 2:** Adjusted association between healthy and sustainable eating efforts and functional nutrition literacy overall and among Mexican Americans, non‐Latino Whites and Mexicans.* International Food Policy Study, 2021.

	All three populations (*n* = 12,079)	Mexican Americans (*n* = 3343)	Non‐Latino Whites (*n* = 2957)	Mexicans (*n* = 5779)
	**AOR (99% CI)** a	**AOR (99% CI)** b	**AOR (99% CI)** b	**AOR (99% CI)** b
Eat more fruits and vegetables				
High likelihood of limited literacy	Reference	Reference	Reference	Reference
Possibility of limited literacy	**1.36 (1.14, 1.62)**	1.31 (0.98, 1.74)	1.28 (0.91, 1.8)	**1.48 (1.16, 1.9)**
High likelihood of adequate literacy	**1.43 (1.20, 1.71)**	**1.51 (1.16, 1.97)**	**1.6 (1.19, 2.14)**	1.26 (0.96, 1.66)
Eat more plant‐based protein sources^c^				
High likelihood of limited literacy	Reference	Reference	Reference	Reference
Possibility of limited literacy	**1.22 (1.02, 1.46)**	0.90 (0.68, 1.19)	0.96 (0.67, 1.37)	**1.48 (1.17, 1.87)**
High likelihood of adequate literacy	1.08 (0.91, 1.29)	0.85 (0.66, 1.10)	0.96 (0.71, 1.3)	**1.30 (1.00, 1.69)**
Eat more whole grains				
High likelihood of limited literacy	Reference	Reference	Reference	Reference
Possibility of limited literacy	**1.22 (1.03, 1.45)**	1.10 (0.84, 1.45)	1.16 (0.82, 1.65)	**1.3 (1.03, 1.63)**
High likelihood of adequate literacy	1.07 (0.90, 1.27)	1.08 (0.84, 1.39)	1.15 (0.86, 1.56)	1.01 (0.78, 1.30)
Eat more protein and fiber				
High likelihood of limited literacy	Reference	Reference	Reference	Reference
Possibility of limited literacy	1.11 (0.93, 1.32)	1.24 (0.93, 1.65)	1.02 (0.73, 1.43)	1.20 (0.94, 1.53)
High likelihood of adequate literacy	1.15 (0.97, 1.37)	**1.44 (1.10, 1.89)**	1.30 (0.98, 1.74)	0.99 (0.75, 1.29)
Eat more organic, ethically sourced, environmentally friendly or locally grown foods				
High likelihood of limited literacy	Reference	Reference	Reference	Reference
Possibility of limited literacy	1.06 (0.89, 1.27)	1.05 (0.79, 1.39)	0.98 (0.70, 1.37)	1.16 (0.91, 1.48)
High likelihood of adequate literacy	0.84 (0.70, 0.99)	0.88 (0.68, 1.14)	0.88 (0.66, 1.17)	0.81 (0.63, 1.06)
Eat less meat, red meat or dairy				
High likelihood of limited literacy	Reference	Reference	Reference	Reference
Possibility of limited literacy	**1.30 (1.09, 1.55)**	1.25 (0.95, 1.65)	1.27 (0.90, 1.80)	**1.39 (1.09, 1.76)**
High likelihood of adequate literacy	1.15 (0.97, 1.37)	1.15 (0.89, 1.49)	1.22 (0.90, 1.64)	1.13 (0.87, 1.48)
Eat less processed products^d^				
High likelihood of limited literacy	Reference	Reference	Reference	Reference
Possibility of limited literacy	**1.40 (1.18, 1.67)**	**1.78 (1.35, 2.35)**	1.43 (1.00, 2.05)	**1.40 (1.11, 1.76)**
High likelihood of adequate literacy	**1.66 (1.39, 1.99)**	**2.73 (2.10, 3.55)**	**2.03 (1.49, 2.79)**	1.27 (0.97, 1.65)
Eat less sugary drinks and 100% fruit juice^e^				
High likelihood of limited literacy	Reference	Reference	Reference	Reference
Possibility of limited literacy	**1.57 (1.31, 1.88)**	**1.83 (1.37, 2.43)**	**1.51 (1.07, 2.12)**	**1.68 (1.30, 2.16)**
High likelihood of adequate literacy	**1.81 (1.51, 2.17)**	**2.72 (2.07, 3.58)**	**2.03 (1.51, 2.72)**	**1.46 (1.10, 1.94)**
Eat less calories, fat, saturated fat, added sugar, and sodium				
High likelihood of limited literacy	Reference	Reference	Reference	Reference
Possibility of limited literacy	**1.46 (1.21, 1.78)**	**1.39 (1.04, 1.87)**	1.35 (0.96, 1.92)	**1.67 (1.26, 2.23)**
High likelihood of adequate literacy	**1.86 (1.53, 2.26)**	**2.28 (1.71, 3.06)**	**1.95 (1.44, 2.65)**	**1.65 (1.20, 2.28)**
Eat less sweeteners				
High likelihood of limited literacy	Reference	Reference	Reference	Reference
Possibility of limited literacy	1.09 (0.91, 1.3)	1.10 (0.84, 1.45)	0.96 (0.68, 1.36)	1.21 (0.95, 1.53)
High likelihood of adequate literacy	1.01 (0.85, 1.2)	0.97 (0.75, 1.25)	1.13 (0.83, 1.53)	0.92 (0.71, 1.20)

*Note:* Bold values indicate significant associations (*p* < 0.01).

* Pooled estimates are shown for all outcomes for completeness; interpretation focuses on population‐specific estimates only when statistically significant interactions were observed.

^a^ Estimates adjusted for population group, age, sex, education level, income adequacy, food security, and language use.

^b^ Estimates adjusted for age, sex, education level, income adequacy, food security, and language use.

^c^ Significant interaction between functional nutrition literacy level and population group. Mexicans differed from Mexican‐Americans and non‐Latino Whites at both possible limited and high likelihood of adequate literacy levels (vs. high likelihood of limited literacy) (all *p*‐values ≤ 0.005).

^d^ Significant interaction between high likelihood of adequate nutrition literacy (vs. high likelihood of limited literacy) and population group, with Mexicans and non‐Latino Whites differing from Mexican Americans, and Mexicans differing from non‐Latino Whites (all *p*‐values ≤ 0.008).

^e^ Significant interaction between high likelihood of adequate nutrition literacy (vs. high likelihood of limited literacy) and population group, with Mexicans differing from Mexican Americans (*p*‐value < 0.001).

In contrast, statistically significant interactions by population were observed for adequate versus limited literacy for self‐reported efforts to eat more plant‐based protein sources, efforts to eat less processed products, and efforts to eat fewer sugary drinks and 100% juices (all *p*‐values ≤ 0.008; Table 2). Population‐specific adjusted models (Table 2) showed that the association between higher functional nutrition literacy levels (vs. high likelihood of limited literacy) and self‐reported efforts to consume more plant‐based protein sources was stronger among Mexicans (range of AOR = 1.30–1.48) than among Mexican Americans (range of AORs = 0.85–0.90) or non‐Latino Whites (range of AORs = 0.96–0.96). For efforts to consume fewer processed foods, having a high likelihood of adequate literacy (vs. a high likelihood of limited literacy) was more strongly associated among Mexican Americans (AOR = 2.73, 99% CI: 2.10, 3.55) than among non‐Latino Whites (AOR = 2.03, 99% CI: 1.49, 2.79) or Mexicans (AOR = 1.27, 99% CI: 0.97, 1.65). For efforts to consume less sugary drinks and 100% juice, the association was stronger among Mexican Americans (AOR = 2.72, 99% CI: 2.07, 3.58) than among Mexicans (AOR = 1.46, 99% CI: 1.10, 1.94).

## 4 Discussion

This study showed that, despite notable sociodemographic differences across populations, Mexican Americans and Mexicans generally reported similar levels of healthy and sustainable eating efforts, but these levels were higher than the levels reported by non‐Latino Whites. Furthermore, higher levels of functional nutrition literacy were associated with reporting greater engagement in most (six out of ten) healthy and sustainable eating efforts across the three populations. However, the strength of some of these associations varied across population groups. Associations between higher literacy and self‐reported efforts to eat less processed products and sugary drinks and 100% juices were stronger for Mexican Americans, while the association with self‐reported efforts to eat more plant‐based protein was stronger among Mexicans.

Despite reporting lower functional nutrition literacy levels, Mexican Americans and Mexicans consistently reported greater engagement in healthy and sustainable eating efforts compared with non‐Latino Whites, who had the highest literacy levels, a pattern consistent with prior evidence linking higher nutrition literacy to White populations and higher socioeconomic position [40, 41, 42]. This seemingly paradoxical pattern (lower literacy yet greater reported engagement in eating efforts) may reflect sociocultural, economic, or structural drivers that operate independently of, or even despite, individual literacy skills. For example, adherence to more traditional dietary patterns characterized by higher consumption of plant‐based foods and minimally processed ingredients, may be more common among Mexican and Mexican American populations, and has been linked to lower levels of acculturation and, in some contexts, to economic constraints that favor less processed, lower‐cost foods [43, 44]. These findings also align with the key messages of the Dietary Guidelines for Americans 2020–2025 [15], as well as with the goals of Mexico's front‐of‐pack warning labels and associated public health communication [37]. As population‐level interventions, these initiatives aim to communicate clear and actionable messages that reduce reliance on individuals' ability to perform detailed numerical interpretations of nutrition information, an ability captured by the functional nutrition literacy measure used in this study, when engaging in healthy eating efforts. In Mexico, continuous healthy eating campaigns have been implemented over the last decade, with the implementation of new front‐of‐pack warning labels being the most recent before data collection. This situation may have contributed to greater awareness and engagement with healthy and sustainable eating efforts. Supporting this interpretation, a five‐country study found that Mexican adults had the highest awareness of front‐of‐package labels and that efforts to reduce non‐recommended nutrients were more strongly associated with use of these labels than with reliance on the traditional nutrition facts label [45].

Among Mexican Americans, cross‐border influences may also play a role. For example, among Mexican Americans analyzed in another study using 2021 data, 88% reported purchasing food from Mexican stores in the prior month, and 64.1% of these participants reported exposure to the Mexican warning labels [46]. Furthermore, many reported that these labels influenced them to avoid purchasing a variety of unhealthy foods at rates that were similar to but lower than what has been found for Mexico [47]. It is also possible that Mexican and Mexican American participants, despite reporting lower levels of functional nutrition literacy, may draw on experiential or culturally transmitted knowledge to guide food‐related decisions. Cultural and familial influences have been identified as important factors shaping eating practices among people of Mexican origin [48], and family support and motivation may also play an important role in dietary self‐management among Mexican Americans [49]. The high burden of diet‐related non‐communicable diseases, particularly diabetes [50], within Mexican‐origin populations may increase exposure to and familiarity with healthy eating messages. Personal and family experiences with these conditions may therefore encourage dietary changes, even among those with limited nutrition literacy. Thus, the greater reported engagement in healthy and sustainable eating efforts among Mexican and Mexican American participants may reflect a combination of cultural, experiential, social, and structural influences that can coexist with lower functional nutrition literacy. In this context, functional nutrition literacy may represent only one of several pathways through which individuals interpret and act on health‐related dietary information.

Beyond differences in the overall prevalence of healthy and sustainable eating efforts, the strength of the association between functional nutrition literacy and specific efforts also varied across populations. Compared with non‐Latino Whites in the US, stronger associations were observed with some efforts among Mexican Americans (efforts to eat more fruits and vegetables and whole grains) and Mexicans (efforts to eat more plant‐based protein sources). The public health and food‐information environment may be particularly relevant when interpreting these differences. In Mexico, for example, communication campaigns implemented in 2020 alongside the new front‐of‐pack warning labels promoted limiting ultraprocessed products and preferring natural and traditional foods, including legumes like beans and chickpeas, as well as maize and vegetables. This policy and communication context provides one possible explanation for the stronger association observed between functional nutrition literacy and self‐reported efforts to consume more plant‐based protein sources among Mexicans. However, although public messaging in Mexico initially aligned with environmentally friendly diets, as mentioned above, sustainability was only recently made explicit with the release of the 2023 Healthy and Sustainable Food Guide [36]. Therefore, national campaigns alone are unlikely to fully explain the patterns observed, particularly those related to limiting the consumption of meat. For Mexican Americans, it is possible that in contexts with higher structural and information barriers, such as the ones faced by this population, functional literacy skills may play a more decisive role in shaping dietary efforts. Future research is needed to further examine these associations. However, because these contextual factors were not directly measured, they cannot explain the observed differences definitively.

Importantly, across all three populations, higher levels of nutrition literacy were consistently associated with reporting efforts to engage in most of the healthy and sustainable eating behaviors studied, including efforts to eat more fruits and vegetables, whole grains, and efforts to eat less meat, red meat or dairy, processed products, sugary drinks and 100% juices, calories, fat, saturated fat, added sugar, and sodium. These findings are in line with evidence from young adults in the US indicating that nutrition literacy was associated with more frequent food label use, which in turn related to better diet quality [51]. In contrast, a systematic review of 18 studies across various countries, including the US, reported no significant associations between health literacy and salt or fat consumption, and findings regarding sugar intake were inconclusive [52]. However, these studies assessed actual dietary intake and the broader construct of health literacy, whereas our outcomes capture self‐reported efforts to modify eating behaviors. Healthy and sustainable eating efforts reflect individuals' self‐reported efforts to adopt healthier and more sustainable dietary practices, rather than objectively measured dietary intake or overall dietary quality. Such efforts may not always translate directly into measurable intake differences, which could partly explain inconsistencies across studies. Thus, our findings suggest that higher functional nutrition literacy may be associated with greater efforts to improve dietary practices, but cannot establish that these efforts translate into actual changes in dietary behavior or dietary quality.

In addition, associations were more consistent for efforts targeting nutrients or ingredients emphasized in public health messaging at the time of data collection (e.g., fruits and vegetables, whole grains, sugar, sodium, saturated fat) [13, 14, 15] than for environmentally oriented behaviors such as eating more plant‐based proteins or reducing meat consumption. This pattern may reflect public health messaging during the study period that did not explicitly incorporate sustainability considerations. In Mexico, sustainability was only formalized within national policy more recently, after the current study was conducted. In 2023, Mexico released the Healthy and Sustainable Dietary Guidelines, which highlight ten recommendations, including consuming local fruits and vegetables, limiting ultraprocessed products and red meat, and increasing intake of plant‐based protein sources [36]. In 2024, the General Law on Adequate and Sustainable Food was enacted, establishing the right to adequate and sustainable food in national policy. This right is defined as access to and consumption of nutritious, sufficient, and culturally appropriate food that meets physiological needs across the life course, strengthens food self‐sufficiency, and takes socioeconomic conditions into account. Additionally, Mexican School Food Standards were updated in 2024 and in 2025 came into force to ban the sale of “junk food” and soft drinks, require schools to offer natural or minimally processed foods and not to offer food preparations high in sugars, saturated fats and sodium, and hold school directors accountable for compliance [53]. In contrast, although the US has implemented updates in School Nutrition Standards, which include the first‐ever limits on added sugars in items like flavored milk, and a gradual reduction in sodium levels through the 2027–2028 school year [54], explicit efforts to address sustainability remain limited. In particular, the 2025–2030 Dietary Guidelines for Americans place substantial emphasis on protein intake, promoting both animal‐ and plant‐based protein foods [15]. Notably, the final guidelines differ from the recommendations of the 2025 Dietary Guidelines Advisory Committee, which placed greater emphasis on plant‐based protein sources and reducing red and processed meat [55]. This shift illustrates how changes in policy priorities can alter the information environment in which individuals make dietary decisions. From a health and sustainability perspective, nutrition messaging should clearly communicate not only the importance of adequate protein, but also the choice of protein sources and their broader implications for health and environmental sustainability. Overall, the results of this study suggest that public health messaging may need to more explicitly integrate sustainability considerations to effectively promote both health and environmental goals.

More broadly, our findings suggest that functional nutrition literacy and public policies may operate synergistically. While nutrition policies, such as front‐of‐package warning labels and school nutrition standards can communicate clear and actionable information and thereby reduce the cognitive load placed on individuals to interpret complex nutrition information, functional nutrition literacy may enhance individuals' ability to understand and act on the information provided by these policies. Thus, strengthening functional nutrition literacy may enhance the potential effectiveness of nutrition policies, whereas well‐designed policies may help reduce disparities associated with differences in individual literacy skills [31, 56], as may be the case with Mexican front of pack warning labels [45]. Although our cross‐sectional data do not allow us to establish such synergistic effects, future studies could help clarify this potential relationship.

By examining Mexican Americans, non‐Mexican Americans, and Mexicans, this study offers a rarely captured cross‐cultural perspective and highlights patterns robust across diverse sociocultural contexts. Few studies have explored the link between health literacy and sustainable eating, so the cross‐country patterns observed here provide a valuable contribution that single‐country studies may overlook. However, results should be interpreted with some limitations in mind, beyond those already addressed. First, the generalizability of the findings may be limited by the sampling method. Despite efforts to recruit low‐education participants in Mexico and the US, the resulting sample is comprised of fewer Mexican Americans and Mexicans with relatively low educational attainment than in the general population. Since functional nutrition literacy is positively associated with education, our sample may overrepresent individuals with higher literacy skills. As a result, we may overestimate the prevalence of eating effort outcomes, given the positive association between nutrition literacy and these behaviors. The measure used to capture functional nutrition literacy, specifically the ability to interpret information from a nutrition facts label, reflects a narrower construct than broader conceptualizations of nutrition or food literacy. It does not capture other relevant dimensions such as nutrition knowledge, attitudes, cultural food knowledge and practices, or food preparation skills. Therefore, our findings should be interpreted as reflecting nutrition label understanding rather than comprehensive nutrition literacy. It is possible that broader forms of nutrition or food literacy may show different or stronger associations with sustainable eating efforts. Finally, we did not assess key constructs (e.g., attitudes, subjective norms, self‐efficacy) from behavior change theories (e.g., Health Belief Model, Theory of Planned Behavior) that may explain the relationship between nutrition literacy and dietary behaviors, but research on such mechanisms should inform future intervention efforts [57].

## 5 Conclusions

Results of this study highlight the diverse ways in which functional nutrition literacy relates to self‐reported dietary efforts across different sociocultural contexts. Overall, Mexican Americans and Mexicans reported more healthy and sustainable eating efforts than non‐Latino Whites, despite having lower levels of nutrition literacy, possibly reflecting the influence of national initiatives promoting healthier diets, particularly in Mexico. At the same time, positive associations between functional nutrition literacy and self‐reported eating efforts across populations suggest that strengthening these skills may help individuals understand and apply public health guidance more effectively. Together, these findings indicate that policies may benefit by combining efforts to improve nutrition literacy with population‐level strategies that reduce reliance on individual interpretation and support healthier choices across diverse populations. The stronger associations observed among Mexican Americans and Mexicans further suggest that functional nutrition literacy may be particularly relevant for supporting healthy and sustainable eating efforts in these populations. Finally, the weaker and less consistent associations observed for environmentally oriented behaviors suggest that public health messaging for all three populations could benefit from more explicitly integrating sustainability considerations to jointly advance health and environmental goals.

## Author Contributions

**Alejandra Jáuregui:** writing – original draft, conceptualization, methodology, formal analysis, investigation, visualization, funding acquisition, writing – review and editing. **Edna Jacqueline Ramirez‐Contreras:** writing – original draft, methodology, formal analysis, writing – review and editing. **Kathia Larissa Quevedo‐Torres:** conceptualization, methodology, writing – review and editing. **Alejandra Contreras‐Manzano:** conceptualization, writing – review and editing. **Christine M. White:** project administration, data curation, methodology, writing – review and editing. **Laura Patricia Arellano‐Gómez:** writing – review and editing. **David Hammond:** funding acquisition, methodology, resources, writing – review and editing. **Rachel E. Davis:** conceptualization, project administration, funding acquisition, writing – review and editing. **James F. Thrasher:** conceptualization, project administration, funding acquisition, writing – review and editing.

## Ethics Statement

The study received ethics clearance through a University of Waterloo Research Ethics Board (REB#30829), the University of South Carolina Institutional Review Board, and the National Institute of Public Health of Mexico (CI: 1641).

## Consent

All participants (adults aged 18–100) provided informed consent before beginning the online survey.

## Conflicts of Interest

David Hammond has provided paid expert testimony on behalf of public health authorities in response to challenges from the food and beverage industry. Other authors have no conflicts of interest to declare. Other authors declare no competing interests.

## Policy on Using ChatGPT and Similar AI Tools

During the preparation of this work, the authors used ChatGPT (OpenAI; GPT‐5.6 Luna) on August 8‐11 to proofread and improve the language and clarity of the manuscript. The tool was used exclusively for language editing and was not used for data analysis, interpretation of results, or generation of scientific content. ChatGPT was used throughout the manuscript, including the Introduction, Methods, Results, and Discussion sections. All AI‐assisted edits were critically reviewed and verified by the authors for accuracy, consistency, and appropriateness, and the authors take full responsibility for the final content of the published article. No sensitive, proprietary, or human participant information was entered into the tool.

## Data Availability

The data that support the findings of this study are available on request from the corresponding author. The data are not publicly available due to privacy or ethical restrictions.
